# Vector-borne and zoonotic diseases of dogs in North-west New South Wales and the Northern Territory, Australia

**DOI:** 10.1186/s12917-017-1169-2

**Published:** 2017-08-15

**Authors:** Amanda J. Shapiro, Graeme Brown, Jacqueline M. Norris, Katrina L. Bosward, Debbie J. Marriot, Nandhakumar Balakrishnan, Edward B. Breitschwerdt, Richard Malik

**Affiliations:** 1 University of Sydney School of Veterinary Science, Building B14, Sydney, NSW 2006 Australia; 2 Department of Microbiology and Infectious Diseases, St. Vincent’s Hospital, Sydney, NSW 2010 Australia; 3 Intracellular Pathogens Research Laboratory, Center for Comparative Medicine and Translational Research, College of Veterinary Medicine, North Carolina State University, Raleigh, NC USA; 4 School of Animal & Veterinary Sciences, Charles Sturt University, Locked Bag 588, Wagga Wagga, NSW 2678 Australia; 5 Centre for Veterinary Education, University of Sydney, Sydney, NSW 2006 Australia

**Keywords:** Dog, *Babesia* spp., *Anaplasma* spp., Haemotropic mycoplasmas, *Coxiella burnetii*, *Bartonella* spp., *Brucella* spp.

## Abstract

**Background:**

Vector-borne diseases of dogs in Australian Aboriginal communities are relatively unexplored. These dogs represent a unique group with variable ecto- and endo-parasitic burdens, nutritional stresses and a general lack of veterinary intervention. We investigated haemoprotozoal and bacterial pathogen prevalences in relation to erythrocyte and platelet numbers in dogs from North-West New South Wales (N-W NSW) and the Northern Territory (NT; Central Australia).

**Methods:**

Real-time PCR (qPCR) amplification of *Anaplasma platys, Babesia vogeli, Mycoplasma haemocanis, Candidatus* Mycoplasma haematoparvum and *Bartonella* spp., serological screening for *Coxiella burnetii,* and *Bartonella* spp. and haematological analyses were performed on dogs from the two cohorts (96 dogs in total). *Brucella suis* serology was determined additionally for the N-W NSW cohort.

**Results:**

*Anaplasma platys* (*n* = 26 dogs)*, Babesia vogeli* (*n* = 7)*, Candidatus* Mycoplasma haematoparvum (*n* = 10 dogs)*,* and *Mycoplasma haemocanis* (*n* = 14) were detected in the sample population (*n* = 96) using qPCR. There were significant associations between (i) *A. platys* and anaemia (OR 8.7, CI 2.4–31.7; *P* < 0.001), thrombocytopenia (OR 12.1, CI 3.4–43.2; *P* < 0.001) and breed (OR 16.1, CI 2.1–121.5; *P* = 0.007), and (ii) between *B. vogeli* and anaemia (OR 11.8, CI 2.3–61.6; *P* = 0.003). Neither protozoal nor bacterial DNA loads, estimated using qPCR, were positively correlated with anaemia or thrombocytopenia. Haemotropic mycoplasmas were not associated with any haematologic abnormality. Four dogs from the NT were seropositive for *Coxiella burnetii*, while no dogs were seropositive for *Brucella suis* or to a panel of *Bartonella* spp. antigens. Despite directed efforts, *Bartonella* DNA was not detected in blood from any of the cohorts studied. A sample of dogs from the NT recruited specifically for *Bartonella* α-proteobacteria growth medium enrichment blood culture were also *Bartonella* PCR negative.

**Conclusions:**

Vector-borne pathogens occur in dogs free ranging near Aboriginal communities, with higher detection rates in NT than N-W NSW. The preponderant haematologic abnormalities were anaemia and thrombocytopenia, likely attributable to *A. platys* and *B. vogeli* infections, but also probably affected by nutritional, parasitic, lactational and environmental stressors. The absence of *Bartonella* spp. is of importance to the Australian setting, and work needs to be extended to tropical coastal communities where fleas are present as well as ticks. Dogs living in and around Aboriginal communities may provide valuable sentinel information on disease infection status of human public health significance.

**Electronic supplementary material:**

The online version of this article (doi:10.1186/s12917-017-1169-2) contains supplementary material, which is available to authorized users.

## Background

Australia is an island continent, with most dogs and people residing in large cities within 70 km of the coast. The population is mainly located in capital cities of the mainland states. This is where most veterinary schools are located, at least until recently when Charles Sturt University (Wagga Wagga, NSW) started a veterinary program. The canine literature is therefore biased towards conditions seen in dogs living in Sydney, Melbourne, Brisbane, Perth, Adelaide and Townsville. For this reason, there is a paucity of information concerning tick-borne disease in Australia, especially diseases transmitted by the brown dog tick, *Rhipicephalus sanguineus*, which is generally found further inland [1]. Limited recent information concerning tick-borne diseases is largely derived from free-roaming dogs living in close association with a limited number of indigenous Aboriginal communities, mainly in the Northern Territory (NT) [2, 3]. A further study concerns vector-borne diseases of pet dogs from Darwin and Southeast Queensland [4, 5].

The interplay of infectious agents transmitted by ticks is relevant firstly to dogs living in areas where these organisms are biological vectors, such as tropical north Queensland, the far north of Western Australia and NT. The concept of ‘One Health’ incorporates intra- and inter- species disease transmission, taking into account environmental variables. Thus, within the current context, dogs represent unique sentinels of infection for human medicine. But infections of dogs in these regions impacts also on other parts of Australia, because people can take dogs where they travel. This might become an even bigger issue if, hypothetically, Darwin was subjected to a tropical cyclone, with subsequent translocation of dogs to other regions. Such a mechanism was implicated anecdotally for the accelerated spread of dirofilariasis in the 1970s following cyclone Tracy [6].

Babesiosis was the first tick-borne disease characterised in Australia by the Veterinary Tropical Health Division, James Cook University in Townsville [7, 8]. *Babesia vogeli* (formerly *Babesia canis vogeli*) is endemic in far north Queensland and the NT. Babesiosis was characterised with the methodologies of the time by Irwin and colleagues [9, 10] and further insights occurred subsequently, as polymerase chain reaction (PCR) testing of venous blood replaced examination of Romanowsky-stained blood films made from capillary blood [11, 12]. PCR simplified testing, being more sensitive and specific than microscopy, with the capacity to detect both clinical and subclinical infections. Canine babesiosis due to *B. vogeli* continues to be a cause of symptomatic haemolytic, subclinical anaemia and thrombocytopenia in these areas [5]. Investigations by various groups have confirmed babesiosis as a common cause of anaemia in NT ‘camp dogs’ in aboriginal communities [3, 5]. Anaemia can be severe enough to cause pale mucous membranes. In puppies and dogs immunosuppressed by malnutrition, life-threatening haemolytic anaemia can develop [9, 13]. Compared to *B. rossi* and *B. canis*, however, *B. vogeli* is considerably less virulent [14, 15], possibly as a result of its longer association with domesticated dogs [16]. Babesiosis due to *Babesia gibsoni* has been reported sporadically in Australia in American pit bulls [17, 18], the disease originating from dogs imported from the USA [19] or Asia [20, 21] and inoculated by fighting, rather than the feeding of ticks [22–24].

In 2001, *Anaplasma platys* was discovered in Australia by Brown and colleagues during an evaluation of the health status of free roaming aboriginal dogs [25]. Further work, including experimental infections, showed *A. platys* caused mild to moderate cyclic thrombocytopenia [26–28], which was usually subclinical. Anecdotally, dogs with heavy *A. platys* infections have been observed to bleed more freely from tick attachment sites than uninfected dogs (Dr Graeme Brown, personal communication). Preliminary data showed some dogs were infected with *B. vogeli*, others with *A. platys*, while a small percentage were infected by both pathogens [3].

Two research groups demonstrated that three or more species of haemotropic mycoplasmas infected free-roaming camp dogs of Central Australia [2, 4, 5, 29]. Two species were preponderant, *Mycoplasma haemocanis* (the more virulent ‘large form’) and *Candidatus* M. haematoparvum with a third unnamed species unable to be characterised further [2], and the fourth found to be *M. haemobos,* a cattle pathogen [5]. The clinical significance of these erythrocytic parasites is currently unclear, as presence of *Mycoplasma* sp. has no apparent correlation with anaemia, even when the cycling threshold (C_*T*_) of the real-time PCR (qPCR) indicates heavy bacteraemia.

The zoonotic pathogen *Rickettsia felis* has recently been shown to have the dog as a definitive host, with transmission requiring fleas or ticks as biological vectors [30]. *Rickettsia felis* has been found in dogs in Southeast Queensland and Central Australia [31]. Other canine tick-borne diseases, such as ehrlichiosis and cytauxzoonosis, are not endemic, but have the potential to be imported from overseas.

*Bartonella* spp. have yet to be reported in the peer-reviewed literature from Australian dogs, even though fleas and ticks are common in many parts of Australia and *Bartonella henselae* and *Bartonella clarridgeiae* are known to be present in fleas [32], foxes [33], humans [34] and cats in Australia, especially young cats [32, 35]. Furthermore, many Australian native animals have a variety of *Bartonella* spp. which could potentially be transmitted to canids [36, 37].

*Coxiella burnetii*, the agent of ‘Q fever’ in human patients and coxiellosis in animals, is a Gram-negative bacterium. Although theoretically capable of being transmitted by fleas and ticks, it is generally transmitted by aerosols from reproductive secretions, or possibly by the ingestion of infectious forms present in uncooked meat or offal from infected animals [38, 39]. A recent Australian serosurvey found dogs living in Aboriginal communities had the highest seroprevalence of coxiellosis [40].

Work to date has emphasised that individual dogs in certain settings can be infected by a combination of flea- and tick-borne infectious agents. Polymicrobial infections with so-called ‘stealth pathogens’ can have complex effects due to intricate interactions such as molecular mimicry and immune-stimulation that cannot be appreciated by studies of a single pathogen under experimental conditions [41, 42]. Thus, in regions where vectors are common, and especially where prophylactic ectoparasitic measures are not undertaken routinely, polymicrobial infections, in association with genetic, nutritional and environmental factors, are likely to impact on haematologic variables and the overall health of infected dogs. Among individuals, disease expression may be influenced by nutritional and environmental stressors, including insufficient quantity or quality of food, pregnancy, lactation or hard work.

The purpose of the present study was to 1) Determine the prevalence of selected zoonotic pathogens and vector-borne pathogens among free-roaming dogs living in Aboriginal communities in different parts of Australia; 2) Assess potential correlations between anaemia and thrombocytopenia and the qPCR C_*T*_ values for *B. vogeli*, *A. platys* and haemotropic mycoplasmas; 3) Determine whether dogs from Central Australia were infected by *Bartonella* spp.

## Methods

### Sample population and specimen collection

Most dogs in Aboriginal communities are of mixed breed and often called ‘camp’ dogs. They are considered hybrids of the dingo (*Canis lupus dingo*) and domestic dogs (*Canis lupus familiaris*). Such dogs are not confined. They may be owned, but are allowed to roam freely or may be strays (recently owned and abandoned). Dogs in this study probably would not roam more than 15 km from where sampled [43]. Because information provided by owners was considered unreliable, estimation of ages was based on dentition. Dogs were classified as ‘pure-bred small’ (1 to 10 kg), ‘purebred medium’ (10 to 20 kg), ‘purebred large’ (> 20 kg) or crossbreds (dingo hybrids). They were classified into four age ranges; 0–1 year, 1–2.5 years, 2.5–6 years and >6 years. Body condition grades (fair, good, excellent; and lactating) were recorded where possible.

**Fig. 1:**
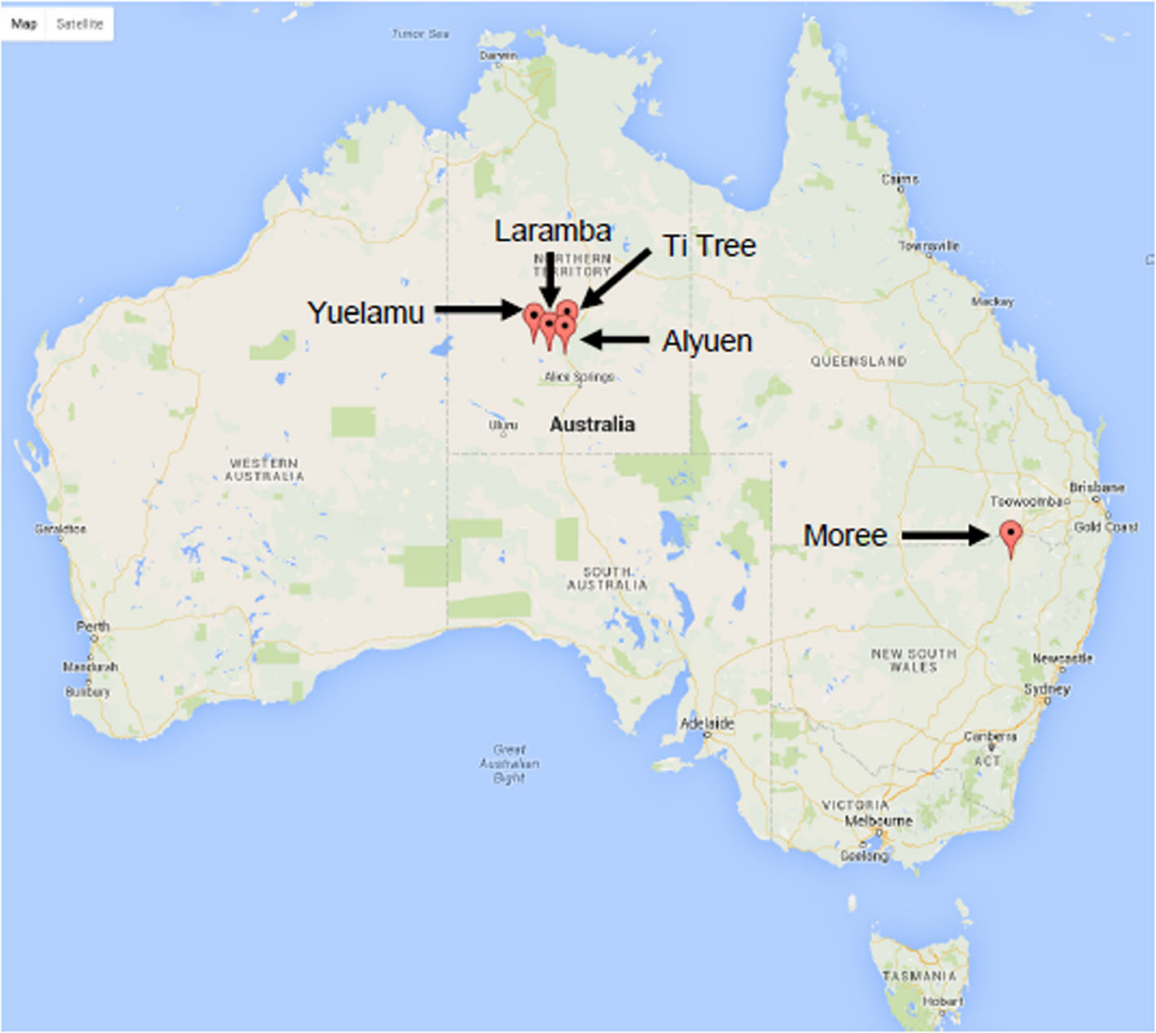
Geographical plot of locations where dogs were sampled. The map identifies the exact locations of the dogs that were sampled (Google Maps 2016)

Dogs were sampled (for haematology and PCR analyses) from two locations; (1) the Ti-Tree communities (NT, Central Australia; *n* = 51) and (2) Moree and nearby districts in N-W NSW, including Mehi Mission (outskirts of Moree), Mungindi, Toomelah and Bogabilla (*n* = 45), while an additional subset of dogs were sampled specifically for specialised *Bartonella* testing) from the Central Australian communities of Yuelamu, Laramba and Alyen (Fig. 1). Dogs lived in and around conventional houses and were either fed by their owners, scavenged leftovers or were able to catch and eat wildlife. All houses in both communities were visited and occupants asked to identify and restrain their dogs. In Moree, dogs in the pound (*n* = 14) were also sampled at the request of the local council ranger; these were in good condition, none had pale gums, a few had sores (most likely resolving bite wounds). All appeared to be ‘town dogs’. Blood from dogs in Ti Tree was collected in November 2010. Blood from Moree dogs was obtained at the end of summer, shortly after drought-breaking heavy rain in February 2013. In August 2014, dogs were sampled from Yuelamu, Laramba and Alyuen specifically in an attempt to determine if *Bartonella* spp. were present in dogs from Central Australia (40 dogs sampled), coinciding with a trip to Australia by EB. Within these communities, each dog was identified and its age and sex determined. The ages of the dogs ranged from approximately 8 weeks to 10 years but because of difficulties encountered in catching and restraining dogs, more adults (> 12 months) than young dogs were sampled. It was noted briefly whether dogs were infested with ectoparasites, or not, and the extent of the infestation.

EDTA anti-coagulated blood and whole blood (clot tube) were collected from dogs, stored at 4 °C in an eski, and transported to the laboratory within 7 days of collection, as soon as was practical. Serum was harvested from whole blood after centrifugation. Residual EDTA blood (after haematologic analyses) and serum were subsequently stored at −80 °C. Samples were collected with the approval of the Animal Ethics Committee of Charles Darwin University (A01019), Westmead Hospital Animal Ethics Committee (AEC protocol number 5073.10.12) and the University of Sydney (N00/11–2006/3/4492). Samples from Yuelamu, Laramba and Alyuen were collected at the request of the Animal Management Coordinator, Central Desert Regional Council.

### Haematology

All blood specimens from the Moree dog cohort were submitted to the University of Sydney’s Veterinary Pathological Diagnostic Services Laboratory (VPDS) for routine haematologic analyses, using automated cell count analysers as well as whole blood smears evaluating differential cell counts and reticulocyte counts. Laboratory reference intervals (RI) for packed cell volume (PCV) and platelet counts (PLT) in dogs are 0.37–0.55 L/L and 200–600 × 10^9^/L, respectively. Blood specimens from the Ti-Tree dog cohort were submitted within 4 days of collection at the closest accessible pathology laboratory, Alice Springs Base Hospital, NT (a human facility). The following parameters were assessed: white cell count, haematocrit, platelet count and mean platelet volume. Reticulocyte counts and differential WBC counts were not performed.

### *PCR analysis of Anaplasma platys, Babesia vogeli, M. haemocanis* and *Candidatus* M. haematoparvum *and Bartonella spp*

EDTA blood was aliquoted, and a portion from each dog was submitted to IDEXX laboratories for multiplex real-time polymerase chain reaction (PCR) testing (Tick/Vector Canine Comprehensive RealPCR™ Panel). Testing was done in their Sacramento, CA laboratory. Results, including C_*T*_ values, were recorded and tabulated.

### Specialised *Bartonella* testing

Following negative results for commercial PCR testing for *Bartonella* spp. (IDEXX laboratories, Sacramento), 40 additional fresh blood samples sourced from Yuelamu, Laramba and Alyuen collected from dogs in August 2014 were shipped to the Intracellular Pathogens Research Laboratory, College of Veterinary Medicine, North Carolina State University, USA for *Bartonella* testing. For serological analyses, *Bartonella vinsonii* subsp. *berkhoffii* genotypes I, II, and III, *B. henselae* Houston-1 strain (H-1), *B. henselae* (San Antonio 2 strain), and *Bartonella koehlerae* antibodies were determined using six indirect immunofluorescence antibody assays (IFA) and fluorescein-conjugated goat anti-human IgG (Pierce Biotechnology, Rockford, IL), as described [44]. To avoid confusion with possible non-specific binding found at low serum dilutions, a cut-off value of 1/64 was used to define a seroreactive titre. Reactive sera at a titre of 1/64 were further tested with 2-fold dilutions out to 1/8192. For molecular detection of *Bartonella* spp. DNA, a previously described approach that includes PCR amplification of *Bartonella* spp. DNA from blood and *Bartonella* α-proteobacterial growth medium (BAPGM) blood cultures at 7, 14 and 21 days of incubation was used [45]. Two *Bartonella* genus and a *B. koehlerae* species-specific PCR (three independent PCR reactions per DNA extraction) were performed using primers designed to amplify 16-23S intergenic transcribed spacer (ITS) region, as described previously [45].

### Indirect immunofluorescent antibody assay (IFA) for serological testing for *Coxiella burnetii*

A modification of a commercial human *C. burnetii* phase I and II specific IFA IgG kit (Vircell, Spain) was used to detect canine IgG antibodies to phase I and phase II *C. burnetii* (Nine Mile strain), as described [46]. Adjustments for canine serum included the use of anti-canine IgG fluorescein isothiocyanate (FITC) conjugate solution (CJ-F-CANG-10ML, Veterinary Medical Research & Development, Pullman, WA, USA) to canine samples. This antibody was used undiluted at the stage when secondary antibody was applied to the control and test wells. All serum samples were tested at the optimised dilution (1/64) and diluent (5% Skim Milk Powder in PBS) [40]. A sample was considered ‘seropositive’ for *C. burnetii* on IFA if it displayed either phase I or phase II (IgG1, IgG2, IgG3) antibodies at a titre of 1/64 or greater. Positive samples were taken to end titre by two fold serial dilutions, beginning at 1/64.

### *Brucella* testing

As swine brucellosis was an emerging infectious disease in northern NSW around the time sampling was conducted in the Moree district, sera from dogs in this subpopulation were also tested using the Rose Bengal Assay (Biomerieux Bucelloslide-Test; Rose Bengal Antigen) at SydPath (St Vincent’s Hospital, Department of Microbiology, Darlinghurst, NSW, Australia) which includes *Brucella* specific agglutinins to *B. melitensis, B. abortus, B. bovis and B. suis*. Two fold serial dilutions of positive serum were used to determine the reciprocal antibody titre. Complement fixation testing for anti-*Brucella* antibodies was conducted at the Elizabeth MacArthur Agriculture Institute, NSW.

### Numerical analysis

Data were analysed with the statistical software GenStat 16.1 (VSN International, Hemel Hempstead, UK), using two sample *t*-tests and logistic regression. Statistical significance was considered at *P* < 0.05. Associations between *A. platys, B. vogeli, M. haemocanis, Candidatus* M. haematoparvum*, C. burnetii, Bartonella spp.* and *B. suis* infection status and potential risk factors (age category, breed, gender, sterilisation status, packed cell volume [PCV] and platelet numbers) were assessed using logistic regression, with odds ratios reported to evaluate impact of factors.

## Results

### *PCR analysis of A. platys, B. vogeli, M. haemocanis and Candidatus* M. haematoparvum *in relation to haematology and population demographics*

The gender, sterilisation status, breed type, age, body condition, presence or absence of anaemia or thrombocytopenia of the study cohort expressed in relation to *A. platys, B. vogeli, M. haemocanis and Candidatus* M. haematoparvum qPCR status and seropositivity to *C. burnetii and B. suis* are presented in Table 1.

**Table 1:** Exposure variables against microbial result. Frequency table representing the variables of gender, sterilisation status, breed type, age, body condition, PCV and platelet count against *Anaplasma platys, Babesia vogeli, Candidatus* M. haematoparvum and *Mycoplasma haemocanis* qPCR positive results, and seropositivity to *C. burnetii, Bartonella spp. and Brucella spp.*

Variables	Category	*A. platys* positive *n* = 26	*B. vogeli* positive *n* = 7	*Candidatus* M. haemato-parvum *n* = 10	*Mycoplasma haemocanis* *n* = 14	*C. burnetii* Ph I/II positive *n* = 4 (1 NT;3 NW-NSW)	*Bartonella spp.* Positive *n* = 0	*Brucella* positive *n* = 0	Total *n* = 96
Gender	Male	12 (23%)	4 (8%)	6 (12%)	8 (15%)	1 (2%)	0	0	52
	Female	14 (32%)	3 (7%)	4 (9%)	6 (14%)	3 (7%)	0	0	44
Entire/Desexed	Entire	24 (35%)	6 (9%)	9 (13%)	8 (12%)	2 (3%)	0	0	68
	Neutered	1 (10%)	0	1 (10%)	2 (20%)	1 (10%)	0	0	10
Breed type	Purebred small	0	0	0	1 (20%)	0	0	0	5
	Purebred medium	1 (5%)	1 (5%)	3 (14%)	5 (23%)	2 (9%)	0	0	22
	Purebred large	0	0	1 (17%)	2 (33%)	0	0	0	6
	Crossbred	25 (40%)	6 (10%)	6 (10%)	6 (10%)	2 (3%)	0	0	63
Age	0–1 year	11 65	4 (24%)	1 (6%)	2 (12%)	1 (6%)	0	0	17
	1–2.5 years	2 (12%)	1 (6%)	2 (12%)	2 (12%)		0	0	17
	2.5–6 years	12 (36%)	0	4 (12%)	2 (6%)	1 (3%)	0	0	33
	> 6 years	0	0	3 (43%)	4 (57%)		0	0	7
Body condition	Excellent	0	0	0	1 (50%)	0	0	0	2
	Good	3 (9%)	2 (6%)	2 (6%)	6 (19%)	2 (6%)	0	0	31
	Fair	23 (46%)	5 (10%)	6 (12%)	6 (12%)	1 (2%)	0	0	50
	Lactating	0	0	0	0	1 (100%)	0	0	1
PCV	Normal	17 (20%)	3 (4%)	10 (12%)	14 (17%)	4 (5%)	0	0	83
	Anaemia	9 (69%)	4 (31%)	0	0	0	0	0	13
Platelet count	Normal	15 (19%)	4 (5%)	10 (12%)	13 (16%)	4 (5%)	0	0	81
	Thrombo-cytopenia	11 (73%)	3 (20%)	0	1 (7%)	0	0	0	15

Overall, dogs were all considered to be in reasonable health (Additional file 1: Figure S1). Some were in suboptimal condition, perhaps as a result of poor nutrition, lactation, equivocal hygiene and less than ideal endo- and ecto-parasticide and vaccination regimens (Additional file 2: Figure S2). Qualitatively, dogs in Central Australia were in less satisfactory condition compared to dogs in N-W NSW (Additional file 3: Figure S3). Dogs from Central Australia had a variable (sometimes large) number of brown dog ticks, some lice but no fleas, while the dogs from N-W NSW rarely had ticks or fleas, and in low numbers.

Anaemia and thrombocytopenia were common findings amongst these dogs, using laboratory reference intervals (RI) determined for healthy dogs living in affluent Australia cities. It was challenging, however, to ascribe anaemia or thrombocytopenia to specific pathogen(s) because the ranges of values for PCV and platelet counts in ‘control’ dogs (i.e. dogs shown to be free of *A. platys*, *B. vogeli* and haemotropic mycoplasmas using real-time PCR testing) were wider than the VPDS RI established for normal healthy dogs.

In dogs shown to be uninfected with the PCR targeted organisms, the wide range of observed values for PCV and platelet numbers was likely attributable to (1) variable, chronic disease states (parasitic, nutritional, recent pregnancy or lactation) and (2) processing delays in relation to blood specimens due to the remote study sites and inevitable delays in testing, which varied from 2 to 4 days depending on the order of sampling. For the ‘control’ (PCR-negative) dogs, the lower limit for the PCV (which defines the presence of anaemia) was taken to be 0.30 L/L (compared to the laboratory RI of 0.39 to 0.50 L/L). For ‘control dogs’, the lower limit of the platelet count was taken to be 60 × 10^9^/L (assuming the highest count and lowest count were likely outliers), rather than 200 × 10^9^/L, the lower limit of normality for fresh blood collected from healthy dogs subjected to atraumatic fast venepuncture and processed within 24 h [47]. The authors acknowledge that there could be other pathogens (e.g. endoparasites) that are unknown and not specifically tested for.

**Fig. 2:**
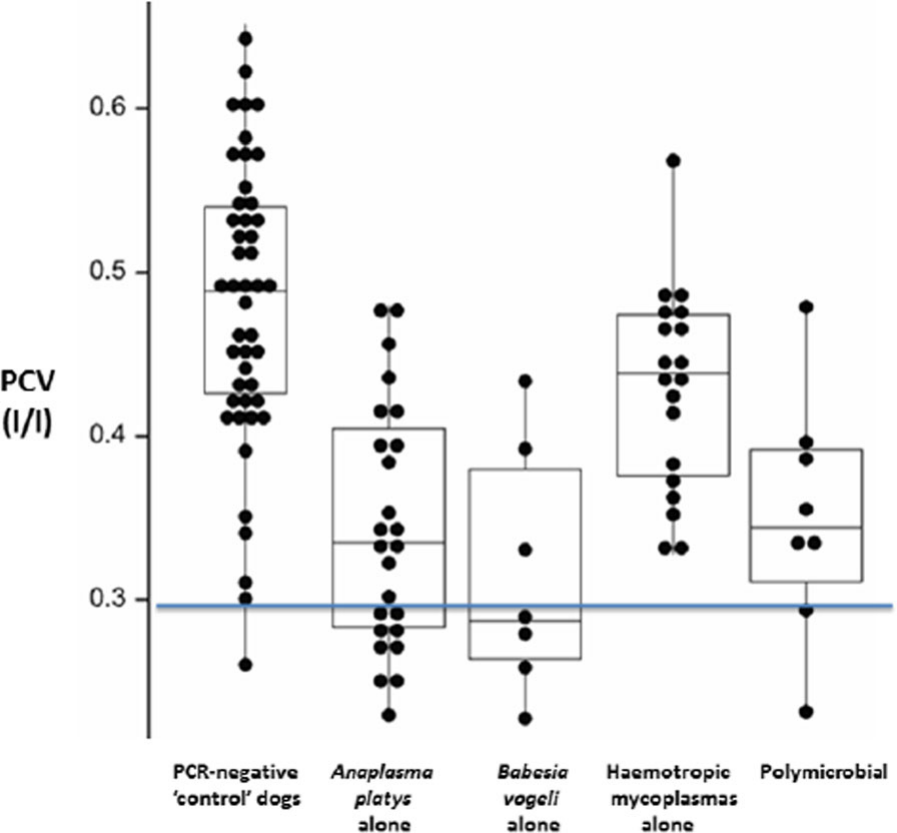
Scatter plots of PCV values from ‘control dogs’ (negative to all pathogenic vector-borne diseases using multiplex qPCR), dogs infected with *A. platys, B. vogeli*, haemoplasmas (one, the other or both species) and polymicrobial infections (various ‘mix-and-match’ combinations of all these tick-borne pathogens). All dots represent an individual dog; box and whisker plots are superimposed (box represents the interquartile range, horizontal line within box represents median value, while the ends of the whiskers represent maximum and minimum values i.e. the range). The RI for PCV values was taken as greater than 0.3 L/L; thus dogs were considered anaemic when the PCV was less than 0.3 L/L (below the blue line)

Inspection of the scatter plots and box and whisker plots illustrates that *A. platys, B. vogeli* and ‘polymicrobial infections’ (combinations of two or more vector-borne pathogens) were commonly associated with anaemia, whereas haemotropic mycoplasma infections (alone) were not (*M. haemocanis* and *Candidatus* M. haematoparvum were combined for simplicity, as preliminary analyses indicated neither alone was associated with anaemia) (Fig. 2). More precisely, 9/25 (36%) dogs that were *A. platys* (only) PCR-positive were anaemic (PCV < 0.30 L/L), 4/7 (57%) of *B. vogeli* (only) PCR-positive dogs were anaemic, 2/9 (12.5%) of dogs with polymicrobial infections were anaemic, while 0/19 (0%) of dogs infected by haemotropic mycoplasmas alone were anaemic. Using two sample *t*-tests, dogs PCR-positive for *B. vogeli* (*n* = 7) were significantly more likely to have a lower PCV than dogs (*n* = 89) negative for this pathogen (*P* < 0.001), dogs PCR-positive for *A. platys* (*n* = 26) were significantly more likely to have a lower PCV than dogs negative (*n* = 70) for this pathogen (*P* < 0.001), while dogs PCR-positive for haemotropic mycoplasmas (*n* = 22) were no more likely to have a lower PCV than dogs (*n* = 74) negative for these pathogens (*P* = 0.981).

**Fig. 3:**
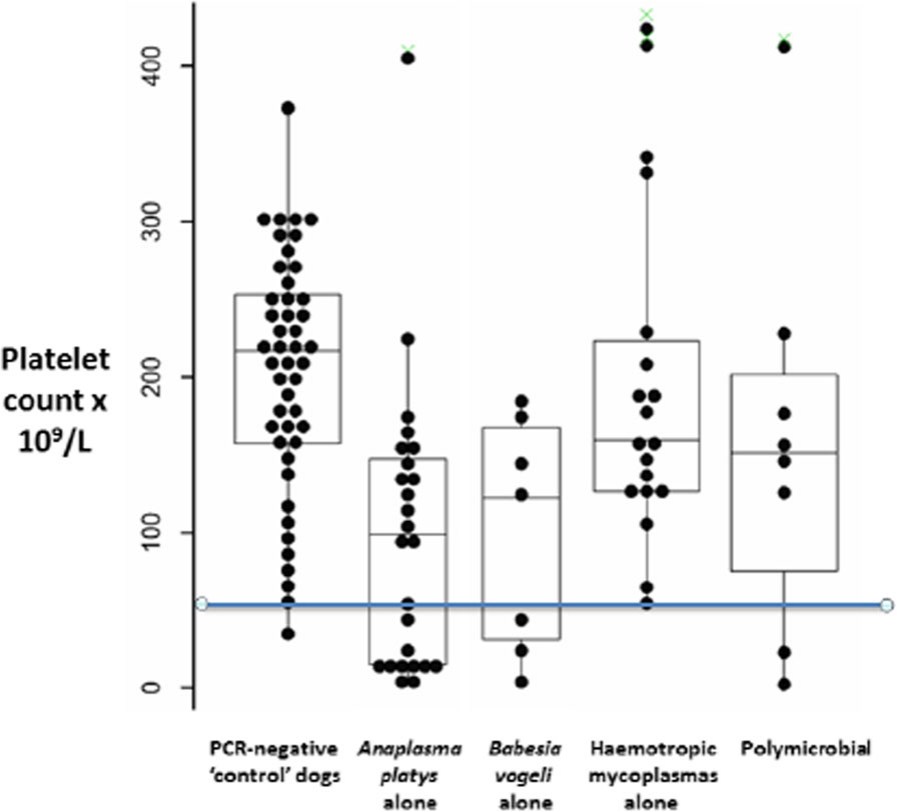
Range of platelet counts from ‘control dogs’ (negative to all pathogenic vector-borne diseases using multiplex PCR), dogs infected with *A. platys, B. vogeli*, haemoplasmas (one, the other or both species) and polymicrobial infections (various ‘mix-and-match’ combinations). All dots represent an individual dog; stem and whisker plots, and outliers, identified by the statistical software package are denoted by a light green cross, are shown. The RI interval for platelet counts was considered to be greater than 60 × 10^9^/L, and thus dogs were considered thrombocytopenic when the platelet count was less than 60 × 10^9^/L (below the blue line)

Similarly, inspection of scatter plots and box and whiskers plots shows that *A. platys, B. vogeli* and polymicrobial infections were commonly associated with thrombocytopenia, whereas haemotropic mycoplasma infections were not (Fig. 3). More precisely, 11/26 (42%) dogs that were *A. platys* (only) PCR-positive were thrombocytopenic (platelet count <60 × 10^9^/L), 3/7 (43%) of *B. vogeli* (only) PCR-positive dogs were thrombocytopenic, 2/7 (29%) of dogs with polymicrobial infections were thrombocytopenic, while 1/22 (5%) dogs with haemotropic mycoplasmas (alone) was thrombocytopenic. Using two sample *t*-tests, dogs PCR-positive for *B. vogeli* (*n* = 7) were had significantly lower platelet counts than dogs (*n* = 89) negative for this pathogen (*P* = 0.04), dogs PCR-positive for *A. platys* (*n* = 26) had lower platelet counts than dogs (*n* = 70) negative for this bacterium (*P* < 0.001), while dogs PCR-positive for haemotropic mycoplasmas (*n* = 22) did not have lower platelet counts than dogs (*n* = 74) negative for these pathogens (*P* = 0.23), although dogs PCR-positive for *Candidatus* M. haematoparvum had higher platelet counts than dogs not infected with this bacterium (*P* = 0.025).

By logistic regression, PCR positivity for *A. platys* was significantly associated with breed (pedigree crossbred animals more likely to be infected; *P* = 0.007), anaemia (*P* < 0.001) and thrombocytopenia (*P* < 0.001), while PCR positivity for *B. vogeli* was associated only with anaemia (*P* = 0.003) (Table 2). There was no association between any infection status and age.

**Table 2:** Logistic regression analysis of variables with a statistically significant association with *A. platys* and *B. vogeli*

Positive	Categories	B	S.E.	Odds ratio	lower 95%	upper 95%	*P* value
*A. platys*	Constant	−1.356	0.271				
	Anaemia	2.167	0.658	8.735	2.407	31.70	<0.001
*A. platys*	Constant	−1.482	0.286				
	Thrombocytopenia	2.493	0.649	12.10	3.392	43.17	<0.001
*A. platys*	Purebred	−3.30	1.00				
	Crossbred	2.78	1.03	16.06	2.124	121.5	0.007
*B. vogeli*	Constant	−3.283	0.588				
	Anaemia	2.472	0.841	11.85	2.281	61.57	0.003

B = Estimate

S.E. = Standard Error

**Fig. 4:**
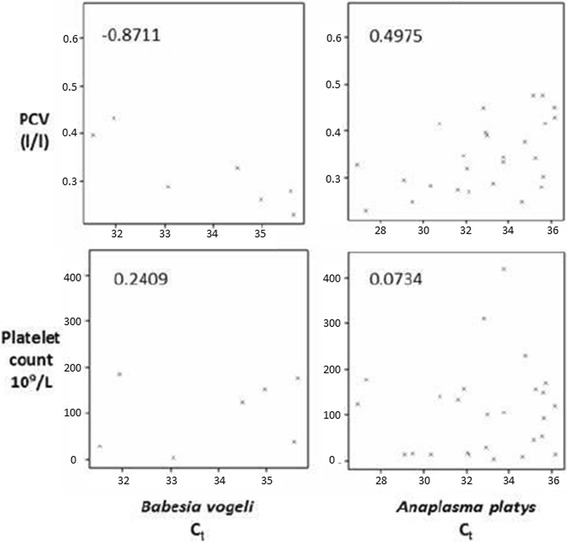
Plots of C_*T*_ versus PCV and platelet count for *B. vogeli* and *A. platys.* Apart from the counterintuitive negative correlation (−0.87) between PCV and *B. vogeli* C_*T*_ values, there was no significant or clinically meaningful correlations evident from these plots

The cycling threshold (C_*T*_) was available from the multiplex qPCR used to ascertain haemoparasitic status of each dog, a high C_*T*_ value indicates a low quantity of the respective pathogen*.* The results were counterintuitive. For *B. vogeli,* there was a negative correlation of −0.87 between C_*T*_ and PCV, inferring that the worse the dogs’ anaemia (i.e. the lower the PCV), the lower the quantity of *Babesia* nucleic acid in their peripheral blood. There was no evidence of a meaningful correlation between C_*T*_ for *Babesia* and platelet numbers and between C_*T*_ for *A. platys* and PCV or platelet numbers (Fig. 4).

### *Bartonella* multiplex qPCR, *Bartonella* serology and BAPGM enrichment culture

The commercial multiplex qPCR assay (IDEXX laboratories, Sacramento) did not detect *Bartonella* spp. DNA in any sample tested. On testing at North Carolina, no dog was seroreactive to any of the six *Bartonella* spp. antigens. Conventional *Bartonella spp.* PCRs using three ITS primer sets from blood and following BAPGM enrichment blood culture for 7, 14 and 21 days were all negative.

### Coxiellosis serological testing

Of 96 dogs tested, four were seropositive for *Coxiella burnetii*. One was an entire male stumpy-tailed cattle dog (guard dog) of unspecified age from the Moree cohort. The remaining three were females: a Staffordshire bull terrier spayed crossbred (5 years-of-age) from Moree, a gravid black kelpie from Moree and an entire juvenile crossbred bitch from Ti-Tree. Further information on these cases has been reported [40].

### Brucellosis serological testing

Only one of the Moree canine cohort tested positive using the Rose Bengal test and it was classed as a weak positive. This dog and all the other dogs from the Moree cohort tested negative on complement fixation testing and are thus considered negative. At the same time as these dogs were tested, a known clinical case of *Brucella suis* infection in a dog with multifocal discospondylitis [48], two dogs with orchitis [47], and two dogs from Yarrabah Aboriginal community near Cairns (Far North Queensland) tested positive using the Rose Bengal test, although the results for the two Yarrabah dogs were flocculating rather than agglutinating.

### Comparison of results between NT and Moree cohorts

Table 3 represents differences in haematologic values and pathogen prevalences between the two geographic areas, NT vs NW-NSW. Differences in prevalence of anaemia alone, thrombocytopenia alone and anaemia plus thrombocytopenia were all significant between the two groups. *Anaplasma platys* was the most prevalent pathogen in the dogs of Central Australia (24/51), with only 2/45 NW-NSW dogs being positive for this pathogen (*P* < 0.001, CI 0.2765–0.5758). There were no significant differences in prevalence of *B. vogeli* and haemotropic mycoplasmas between the two subpopulations. The presence of co-infections was also significantly different between the groups (*P* = 0.042, CI 0.01123–0.2188).

**Table 3:** Summary data concerning anaemia, thrombocytopenia, *B. vogeli, A. platys,* haemotropic mycoplasmas and co-infections in the two different areas tested, as well as *P* values for the two sample binomial *t*-tests comparing the two cohorts

Aboriginal Community	Haematology		PCR				
	Anaemia	Thrombocytopenia	Anaemia and thrombocytopenia	*Babesia vogeli*	*Anaplasma platys*	Haemotropic mycoplasmas	Co-infections^a^
NT (*n* = 51)	13 (26%)	14 (27%)	9 (18%)	5 (10%)	24 (47%)	12 (24%)	7 (14%)
NW-NSW (*n* = 45)	0	1 (2%)	0	2 (4%)	2 (4%)	10 (22%)	1 (2%)
Total	13	15	9	7	26	22	8
*P* value	< 0.001	< 0.001	0.003	0.314	< 0.001	0.879	0.042

^a^The co-infections for the 7 NT dogs consisted of *A. platys* and *B. vogeli* (2 dogs), *A. platys* and *B. vogeli* and *Mycoplasma haemocanis* (1 dog), *A. platys* and Candidatus *M. haematoparvum* (2 dogs), *A. platys* and *M. haemocanis* (2 dogs). The co-infections in the 1 NW-NSW dogs consisted of *A. platys* and *B. vogeli*

## Discussion

Within the two study cohorts, there were conspicuous differences in the prevalence of tick-borne diseases, with all three genera (*Anaplasma, Babesia,* haemotropic *Mycoplasma*) of tick-borne pathogens being less common in NW-NSW than Central Australia. This was correlated with both a much lower prevalence of ticks and also better nutrition and overall health status. The results of this study build on previous work concerning vector-borne and zoonotic infectious diseases of free living dogs in and around Aboriginal communities [2–5, 25, 31, 49, 50].

There was a statistically significant association between the presence of *B. vogeli* and/or *A. platys* and the presence of anaemia and/or thrombocytopenia. Having said this, in the great majority of dogs, the deficiency in red cell mass and platelet numbers was minor, and unlikely to give rise to clinical disease. This is in accordance with the literature, where *B. vogeli* is considered less virulent than its two sibling species and tends to produce subclinical disease, except in young or immunosuppressed dogs [9, 13, 15, 16]. *A. platys* strains in Australia are considered minimally pathogenic, although in some dogs, especially younger dogs with co-infections with other organisms, thrombocytopenia might be severe enough to impact patients subjected to injury or surgery, e.g. dog fights or surgical neutering [3]. It might be prudent therefore to evaluate a peripheral blood smear in the field to ensure platelet numbers are adequate, or to perform a platelet function test e.g. buccal mucosal bleeding time prior to major surgery [51]. Intra-operative bleeding has not been reported as a clinical problem in the field during large-scale surgical neutering campaigns in these communities. It has been the experience of one of the authors (GB) that removing ticks is a good platelet function test, dogs with *A. platys* often having prolonged bleeding from tick attachment sites. It was of great interest to observe that there was no positive correlation, and indeed in one instance (*B. vogeli* and anaemia) there was a negative correlation, between the quantity of bacterial or protozoan DNA in blood and red cell mass or platelet count (Fig. 3). In the instance of *B. vogeli*, the simplest explanation for this finding would be that when anaemia occurs in the setting of long-standing subclinical infection, anaemia is due to sequestration of infected erythrocytes by the mononuclear phagocytic system, reducing or eliminating parasitised red cells and the organisms they contain from peripheral blood.

Although commonly encountered, haemotropic mycoplasmas did not impact on haematologic values in a clinically meaningful fashion. This is also in accord with the traditional view from the literature, which suggests that in the absence of splenectomy or immunocompromise, neither *M. haemocanis* nor *Candidatus* M. haematoparvum causes haemolytic anaemia [4, 5, 52–54].

The most striking observation of the present work was an unequivocal negative finding: PCR testing of whole blood, even after a special pre-incubation step using BAPGM media (in one subgroup), failed to detect *Bartonella* nucleic acid from any dog in either of the two sites tested. Likewise, there was no serological evidence for previous *Bartonella* infection. Although dogs around the world can be infected with numerous *Bartonella* spp., *B. henselae, B. vinsonii* subsp. *berkhoffii* and *B. koehlerae* were most frequently documented in sick dogs in the United States [55]. Following the initial isolation of *Bartonella vinsonii* subsp. *berkhoffii,* tick exposure was determined to be a risk factor for prior exposure based upon IFA seroreactivity [56]. Based upon the high prevalence of both *Ehrlichia canis* and *B. vinsonii* subsp. *berkhoffii* antibodies in dogs from the south-eastern United States [56], *R. sanguineus* was a suspected vector, although this has never been confirmed [57]. Similar to the results of this study, a subsequent investigation from Brazil failed to identify an association between *E. canis* serology or PCR positivity and *Bartonella* spp. antibodies [58]. However, as recent research has documented at least four phylogenetically distinct clades (that justify different species designation) within the morphological designation *R. sanguineus* [59], a role for *Bartonella* spp. transmission for ‘*R. sanguineus’* clades around the world requires further study.

Cats are underrepresented as companion animals in most Aboriginal communities, certainly in the ones sampled in Central Australia. So the likelihood of dogs contacting any of the feline-adapted *Bartonella* (*B. henselae, B. clarridgeiae, B. elizabethiae*) via cat fleas would be minimal, especially in a hot, dry, arid environment where fleas are rare to absent, while brown dog ticks are conspicuously common. Furthermore, *B. vinsonii* subsp. *berkhoffii* has yet to be reported from any Australian jurisdiction, possibly because Australia does not have wolves or coyotes, the natural reservoir for this organism in North America [60–62]. In the context of sick dogs, both IFA serology and PCR from blood lack diagnostic sensitivity as compared to BAPGM enrichment blood culture [55]. In addition, because *B. henselae, B. vinsonii* subsp. *berkhoffii* and *B. koehlerae* strains in Australia might differ antigenically from North American strains used for testing in this study and would be unlikely to cross react with indigenous Australian marsupial strains [44, 63], negative serology results should be viewed with caution. These factors could account for the absence of published reports of canine bartonellosis from Australia, despite the widespread availability of multiplex PCR assays.^1^ It is important that studies such as this be extended to regions where fleas are more common and dogs encounter more than one tick species, such as the coastal parts of tropical north Queensland, because in this environment *Bartonella* spp. may be more likely to ‘spill over’ from cats and foxes to dogs.

It is quite difficult to directly compare the work presented here with that done previously in Australia, as the communities sampled were somewhat different, as were the methodologies employed, including the specifics of the PCR assays. Without equivocation, Traub’s group has shown dogs from central Australian indigenous communities had much higher prevalence of all tick-borne pathogens than dogs in Darwin, which in turn had higher overall prevalences than south east Queensland and Sydney [4, 5]. Presumably this reflects the better condition of owned dogs in Darwin, with better ectoparasitic control, nutrition and so forth, while in Brisbane and its environs and Sydney, *Rhipicephalus* is just not sufficiently common to be an effective disease vector, the hard tick *Ixodes holocyclus* being the preponderant tick species. It should be noted that our ‘healthy’ dogs from the NT living in indigenous communities were, on the whole, more affected by tick-borne pathogens than the ‘affected’ cohort of hospital patients in the 2015 study by Hii et al. [4].

Dogs in indigenous communities sampled were generally judged to be healthy, despite the commonness of tick-borne diseases. The presence of tick-borne pathogens causing subclinical anaemia and thrombocytopenia complicates the diagnosis of diseases common in owned dogs (such as primary immune-mediated haemolytic anaemia [IMHA] and thrombocytopenia [IMT]) in nearby regions such as Alice Springs and Darwin, as there is some flow of dogs between cities in the NT and the surrounding communities. The response to specific anti-infective therapy might be required to dissect out the contribution of various infectious agents.

In dogs living in indigenous communities, the high prevalence of tick-borne diseases requires addressing, even though the majority of dogs in our study appeared in reasonable condition. Our view is that the regular application of acaricides, such as topical moxidectin (especially inexpensive off-label ‘pour on’ cattle formulations), represents a key control measure, perhaps combined with environmental treatment to reduce the number of adult and juvenile ticks, so that immunologically naïve pups are not subjected to sudden inoculation. It is difficult to envisage how imidiocarb or doxycycline could be routinely given in an indigenous setting, because of the requirement to treat dogs for several consecutive days with painful injections or tablets, respectively. If dogs from Aboriginal communities become translocated into pet homes in places like Alice Springs or Darwin, haematologic and multiplex qPCR testing would be prudent, so that subclinical carriers of these blood-borne pathogens could be treated to either eliminate the infection, or more realistically, reduce the extent of the infection so that clinical manifestations are unlikely to develop and transmission to dogs in non-endemic areas is minimised. Likewise, blood donors in such geographies should be tested by multiplex qPCR, as transfusion of blood containing these usually mild pathogens might cause more substantive issues if given to patients with IMHA and/or IMT, where immunosuppressive therapy will be on-going, or after splenectomy.

This study incorporated a ‘One Health’ perspective as bartonellosis, coxiellosis and brucellosis are all important zoonoses, with dogs from Aboriginal communities potentially serving as sentinels for these infections. Bartonellosis has been discussed at length already. In relation to coxiellosis, 4/96 dogs tested sero-positive for *C. burnetii* infection. None of these was symptomatic, but worryingly two of the dogs were sexually intact females, including a pregnant bitch. Despite the apparently high prevalence of anti-*Coxiella* antibodies in dogs living around indigenous communities, Q fever is not considered a common or important disease of Aboriginal people, despite the very close relationship they have with their dogs [40]. One of the seropositive dogs in the NW-NSW cohort was actually a kelpie belonging to a drover, rather than residing in an Aboriginal community. The Moree region subpopulation was also tested for serological evidence for subclinical brucellosis. Only one dog tested weak-positive with the Rose Bengal method, but as it was negative on complement fixation testing, it was classed as negative as per laboratory protocol, although the dog could remain subclinically infected or have eliminated the infection resulting in a residual antibody titre. From a ‘One Health’ perspective, this report importantly draws light to both negative and positive dog data which are equally instructive for both canine and human health.

## Conclusions

The vector-borne pathogens *B. vogeli*, *A. platys* and haemotropic mycoplasmas were prevalent in dogs living in Aboriginal communities in Central Australia, but less common in similar environments in NW-NSW. Although a minimally pathogenic bacterial infection in dogs and humans, the potential for *A. platys* infection should be investigated among sick human individuals in indigenous communities, due to previously mentioned reports in humans overseas. *B. vogeli* was associated with anaemia, and to a lesser extent thrombocytopenia, *A. platys* was strongly associated with thrombocytopenia, whereas neither haemotropic mycoplasma was associated with any haematologic aberration. Similar to the dogs in this study, co-infection with *A. platys* and *Candidatus* M. haematoparvum was reported in a veterinarian, who was concurrently infected with *B. henselae* [64]*.* Polymicrobial combinations of these infectious agents were common in dogs, yet they had little discernible clinical impact. Serological evidence for coxiellosis was evident in one male and three female dogs, yet spill over into in-contact humans in these geographical regions is unknown. The presence of tick-borne pathogens in dogs residing or sourced from these indigenous communities must be considered when investigating haematologic disorders such as anaemia and thrombocytopenia. It remains problematic to differentiate between primary IMHA or IMT from disease secondary to the immune response to these pathogens, or to the actions of the pathogens themselves. Testing of blood donors in areas endemic for these blood-borne pathogens is mandatory, to prevent infection of naïve patients.

## Additional files

Additional file 1: Figure S1. Physical appearance of dogs from Yuendumu. The condition of dogs in Central Australia, NT (a) was lower than those dogs from Moree, N-W NSW (b), yet overall their body condition scores were seen as fair. (Images with permission and courtesy of Dr. Graeme Brown). (PNG 716 kb)

Additional file 2: Figure S2. Pups from Yuendumu, Central Australia. Condition of young pups (a) are poor to fair, with clear visibility of ribs and ‘tucked up’ appearance of abdomen. Some dogs are in better condition than others (b). Landscape is seen as typical red sandy soil, with signs of erosion and negligible grass or plant cover. (Images with permission and courtesy of Dr. Graeme Brown). (PNG 701 kb)

Additional file 3: Figure S3. Image of dog sampled in Moree distinct. The different geographical appearance of the landscapes in Moree and Ti Tree are evident from this picture. The dog appears to be a pedigree hybrid type with robust physical appearance in contrast to dogs from the NT. (PNG 671 kb)

Additional file 4: Dataset upon which publication conclusions rely. (XLSX 24 kb)

## Abbreviations

BAPGM: Bartonella α-proteobacteria growth medium
C_*T*_: Cycling threshold
DNA: Deoxyribonucleic acid
EDTA: Ethylenediaminetetraacetic acid
IFA: Indirect immunoflourescent antibody assay
IMHA: Immune-mediated haemolytic anaemia
IMT: Immune-mediated thrombocytopenia
NSW: New South Wales
NT: Northern Territory
PCR: Polymerase chain reaction
PCV: Packed cell volume
qPCR: Real-time PCR
RI: Reference intervals

## Footnotes

1 The authors are aware of an unpublished case of *Bartonella henselae* infection in a 10-year-old ‘Blue Heeler’ in July 2014. The dog’s major clinical problem was severe thrombocytopenia. Blood was tested by Idexx; the multiplex PCR was negative for *Babesia*, *Anaplasma*, and haemotropic mycoplasmas but positive for *Bartonella* and typed as *B. henselae* based on sequence analysis. The dog was initially treated empirically with doxycycline and two treatments of imidiocarb, 2 weeks apart. It was then given a combination of doxycycline and enrofloxacin and made a full recovery. It was retested for *Bartonella* 2 months later and was PCR-negative. Given that qPCR directly from blood is an insensitive means for detecting bartonellosis in dogs, horses and humans, negative results are not surprising. It is likely more dogs in Australia have had clinical bartonellosis.
