# Supplementary figures and images for: Vector-borne and zoonotic diseases of dogs in North-west New South Wales and the Northern Territory, Australia
Source: BMC Vet Res. 2017 Aug 15;13:238. doi: 10.1186/s12917-017-1169-2 (PMC5558717; doi:10.1186/s12917-017-1169-2)

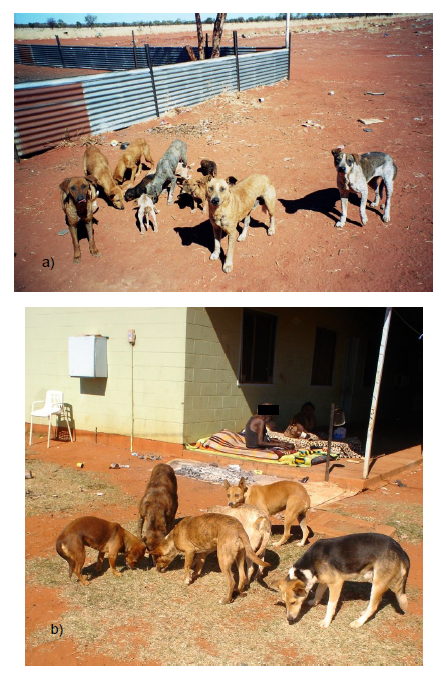

Supplement: Supplementary file 1 — Physical appearance of dogs from Yuendumu. The condition of dogs in Central Australia, NT (a) was lower than those dogs from Moree, N-W NSW (b), yet overall their body condition scores were seen as fair. (Images with permission and courtesy of Dr. Graeme Brown). (PNG 716 kb) [file 12917_2017_1169_MOESM1_ESM.png]

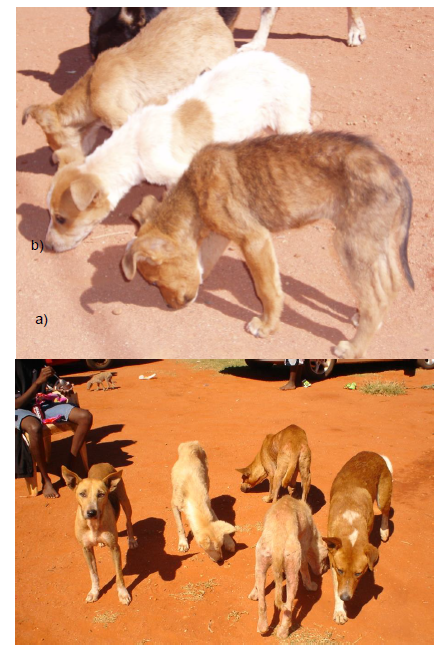

Supplement: Supplementary file 2 — Pups from Yuendumu, Central Australia. Condition of young pups (a) are poor to fair, with clear visibility of ribs and ‘tucked up’ appearance of abdomen. Some dogs are in better condition than others (b). Landscape is seen as typical red sandy soil, with signs of erosion and negligible grass or plant cover. (Images with permission and courtesy of Dr. Graeme Brown). (PNG 701 kb) [file 12917_2017_1169_MOESM2_ESM.png]

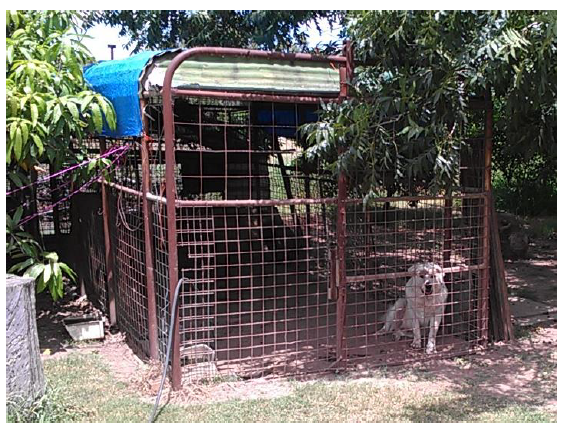

Supplement: Supplementary file 3 — Image of dog sampled in Moree distinct. The different geographical appearance of the landscapes in Moree and Ti Tree are evident from this picture. The dog appears to be a pedigree hybrid type with robust physical appearance in contrast to dogs from the NT. (PNG 671 kb) [file 12917_2017_1169_MOESM3_ESM.png]
